# Intracellular delivery of therapeutic proteins. New advancements and future directions

**DOI:** 10.3389/fbioe.2023.1211798

**Published:** 2023-05-25

**Authors:** Ilaria Porello, Francesco Cellesi

**Affiliations:** Department of Chemistry, Materials and Chemical Engineering “G. Natta”, Politecnico di Milano, Milan, Italy

**Keywords:** intracellular delivery, therapeutic proteins, protein delivery, polymeric nanocarriers, cellpenetrating peptides, protein resurfacing

## Abstract

Achieving the full potential of therapeutic proteins to access and target intracellular receptors will have enormous benefits in advancing human health and fighting disease. Existing strategies for intracellular protein delivery, such as chemical modification and nanocarrier-based protein delivery approaches, have shown promise but with limited efficiency and safety concerns. The development of more effective and versatile delivery tools is crucial for the safe and effective use of protein drugs. Nanosystems that can trigger endocytosis and endosomal disruption, or directly deliver proteins into the cytosol, are essential for successful therapeutic effects. This article aims to provide a brief overview of the current methods for intracellular protein delivery to mammalian cells, highlighting current challenges, new developments, and future research opportunities.

## 1 Introduction

In the last years, protein-based therapeutics have gained an increasing interest in all areas of medicine (Lv et al., 2019; Zhang S. et al., 2020), attracting the attention of the major pharmaceutical industries (Ren et al., 2022; Pandya and Patravale, 2021), due to their remarkable potentials for treatment, diagnosis, and even prevention (Pakulska et al., 2016; Sá et al., 2021; Tan et al., 2021) of several human pathologies (Liu et al., 2022). Protein therapeutics show notable pharmacological efficacy (Pakulska et al., 2016; Liu X. et al., 2019) combined with high therapeutic potency and selectivity with respect to traditional low molecular weight drugs (Cheng, 2021). Compared to small synthetic molecules (Mitragotri et al., 2014; Slastnikova et al., 2018), proteins offer the advantage to be active and effective at lower concentration with high substrate specificity, favoring minimal adverse effects (Leader et al., 2008) and reduced risks of off targets (Hou et al., 2016; Gao et al., 2018).

Previous studies show that most attractive targets are typically located inside the cell (Postupalenko et al., 2015; Tan et al., 2022), thus, in order to exploit full potential of protein-based therapeutics, intracellular protein delivery is fundamental to target intracellular biomolecules (Gu et al., 2011; Mitragotri et al., 2014; Scaletti et al., 2018; Liu X. et al., 2019; Lv et al., 2019). This represent one of the major challenges to overcome since proteins are large and complex biomolecules (Lee et al., 2019; Goswami et al., 2020; Raman et al., 2021; Davis et al., 2022), with markedly hydrophilic features (Lv et al., 2020), resulting in poor cell membrane permeability (Postupalenko et al., 2015; Wang and Yu, 2020). Hence, the not spontaneous crossing of the anionic-hydrophobic cell membrane (Mulgrew-Nesbitt et al., 2006) will limits the currently marketed protein drugs to extracellular targets (Marschall et al., 2014; Mitragotri et al., 2014; Slastnikova et al., 2018; Gaston et al., 2019; Qin et al., 2019).

The objective of this concise review is to outline the existing techniques for delivering proteins inside mammalian cells, aiming to highlight the current challenges, recent advancements, and potential research prospects in this field.

## 2 Developments and challenges in intracellular protein delivery

Different exogenous proteins have been recently explored for intracellular delivery, to modulate cell function and fate, by targeting disease-relevant intracellular receptors. Various strategies for intracellular targeting of antibodies, their fragments, or antibody-like molecules have been extensively reported in other works (Stewart et al., 2016; Slastnikova et al., 2018; Xie et al., 2020). Due to their remarkable specificity and affinity for a target molecule, antibodies are widely used for inhibiting specific activity and for diagnostics, as well as for basic experimental tools, given their role in unveiling cell signaling pathways and diseases mechanisms. Moreover, other therapeutic proteins have been investigated for targeting intracellular sites, including systems for genome editing, induction of apoptosis or toxicity, and blocking specific protein expression, as summarized in Table 1.

**TABLE 1:** Examples of therapeutic proteins with intracellular target.

Therapeutic protein		Advantages	Cells/Animal model	References
Clustered regularly interspaced short palindromic repeat-associated nuclease Cas9	CRISPR-Cas9	Gene editing	Human U2OS cells, T-cell	Zuris et al. (2015), Wang et al. (2016), Stadtmauer et al. (2020)
CRISPR-Cas9-single guide RNA complex	CRISPR–Cas9-sgRNA	Gene editing	Human U2OS-EGFP cells, U2OS-EGFP xenograft tumors in nude mice	Sun et al. (2015)
Transcription activator-like effector nuclease	TALEN	Gene editing	HEK 293T cells, human T-cell	Zuris et al. (2015), Stadtmauer et al. (2020)
Antigen from enterovirus 71	VP_1_	Cellular vaccines	BALB/c mice	Qiao et al. (2018)
Protein phosphatase 1B	Ppase 1b	Suppresses tumor necrosis factor-α-induced systemic inflammatory response	HEK 293T cells, mouse model	Yu et al. (2021)
Ribonuclease A	RNase A	Toxic effects in cells	MSC, CD4^+^ T-cell, cancer cells, HeLa cells	Wang et al. (2014), Liew et al. (2020), Barrios et al. (2022)
Saporin	Sap	Blocks the synthesis of proteins in cells	MSC, CD4^+^ T-cell, cancer cells	Wang et al. (2014), Barrios et al. (2022)
Cre recombinase	Cre	Induce site-specific DNA recombination	HEK cells, HeLa cells, MDA-MB-31 cells, RAW 264.7 cells, mammalian cells, HEK 293T cells	Cronican et al. (2010), Kaczmarczyk et al. (2011), Zuris et al. (2015), Goswami et al. (2023)
Caspase-8	CASP8	Apoptosis-inducing protein Susceptible to inactivation during delivery process	HEK 293T cells	Kaczmarczyk et al. (2011)
TRAIL protein	TRAIL	Amplify apoptotic signal	Cancer cells	Sun et al. (2016)
Caspase 3	CASP3	Apoptosis-inducing protein Susceptible to inactivation during delivery process	HeLa cells	Tang et al. (2013), Ventura et al. (2015)
TRAIL Apo2 ligand	TRAIL-Apo2	Cytotoxic protein	C6 glioma cells	Prasetyanto et al. (2016)
Onconase	Onc	Cytotoxic protein	C6 glioma cells	Prasetyanto et al. (2016)

The clinical applications of these protein drugs face several limitations in terms of delivery efficacy, stability, and final intracellular activity. Additional obstacles, such as fast enzymatic degradation in the bloodstream (Yan et al., 2022) and possible immune system response [common to therapeutic proteins for extracellular delivery (Parodi et al., 2017; Moncalvo et al., 2020)], must be considered.

Although delivery vehicles can help transporting proteins across cell membranes (Luther et al., 2020), the limited number of binding sites on protein surface represents a key issue that hinders the efficient transport of the cargo proteins by the appropriate carrier (Lv et al., 2020). In fact, the surface of proteins is notoriously heterogeneous, being covered by cationic, anionic, and hydrophobic groups. For this reason, carriers often rely on covalent conjugation of functional molecules (Loibl and Gianni, 2017), although critical disadvantages of such systems include the limited availability of residues for conjugation, potential effects on protein folding and function (Weiner, 2015) [given their sensitivity to chemical modifications (Zhang et al., 2018)], and complex workflow steps. Moreover, cellular internalization often brings the nanocarrier to the cytoplasm via endosomes, by means of naturally occurring endocytosis processes, such as clathrin-mediated endocytosis (Kaksonen et al., 2006), caveolae-mediated endocytosis (Nabi and Le, 2003) or micropinocytosis (Kerr and Teasdale, 2009). Endosomes will ultimately be transformed into lysosomes, with a consequent increase of the environment acidity and the secretion of various proteases (Niamsuphap et al., 2020), causing protein degradation. Nonetheless, endosomal discharge is generally an inefficient process, with only ∼1% of the total cargo being released intact into the cytoplasm excluding deterioration or expulsion by exocytosis (Stewart et al., 2016). Non-specific clearance by the reticuloendothelial system (RES) after systemic administration of protein-loaded carriers generally causes a significant decrease of the delivery efficiency into the target tissues. To address this issue, strategies as a transient stealth coating of liver reticuloendothelial cells by two-arm-PEG-oligopeptide may be effective in preventing the clearance of nonviral and viral nanovectors by the liver sinusoidal endothelium (Dirisala et al., 2020).

Therefore, the development of efficient and versatile delivery strategies is crucial for an effective use of protein drugs (Feng et al., 2022). They need to reach cytoplasmic targets safely (Wang and Yu, 2020) by encapsulating the desired cargo into cell-degradable nanocarriers (Tsao et al., 2020; Liu et al., 2022), able to trigger endocytosis and endosomal disruption (Zhang et al., 2018), or capable to directly deliver proteins into the cytosol (Sun et al., 2022).

## 3 Intracellular protein delivery techniques: An overview

**FIGURE 1:**
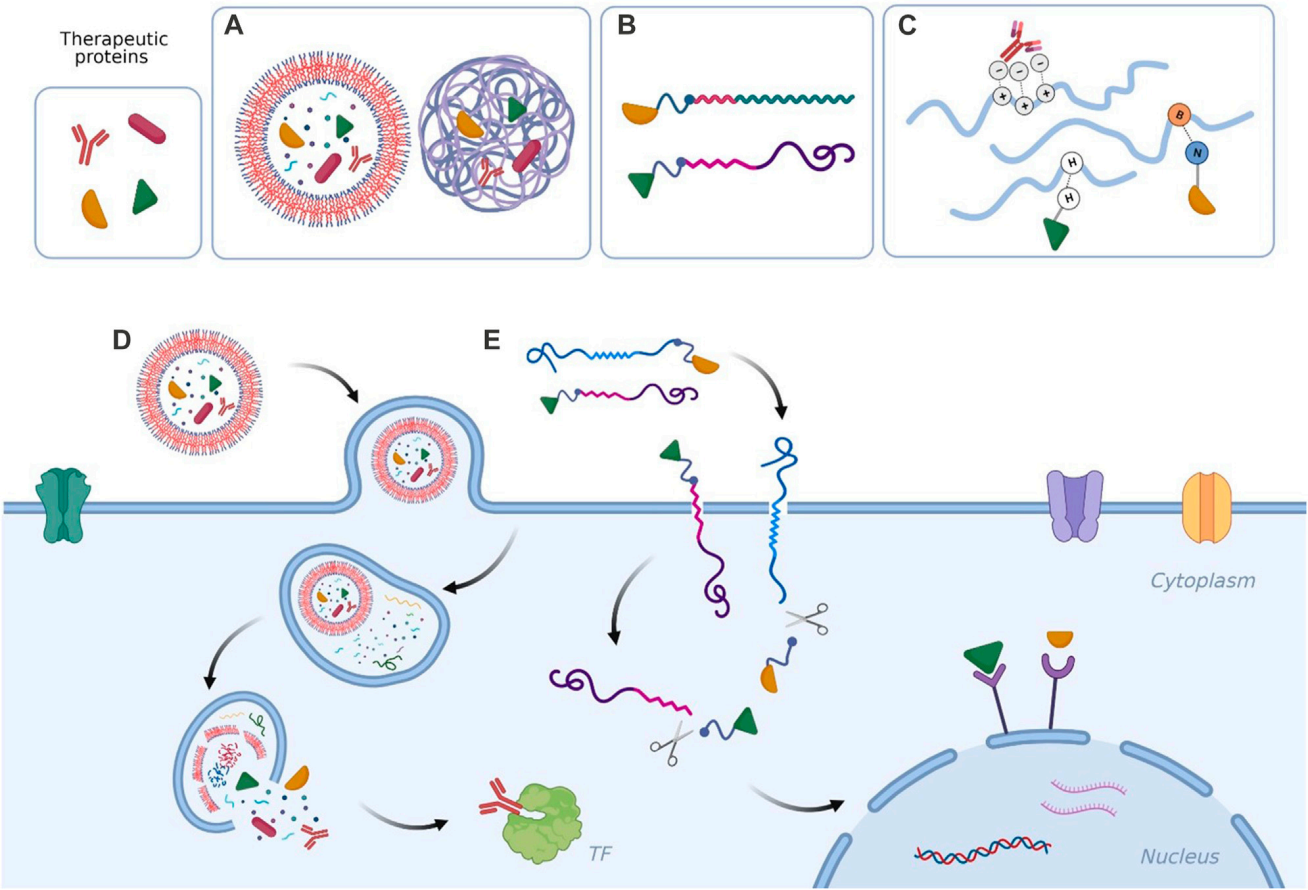
Examples of **(A)** therapeutic proteins encapsulated in polymersomes and in polymeric nanoparticles; **(B)** therapeutic proteins conjugated to amphiphilic polymers and to cell-permeable peptides; **(C)** therapeutic proteins forming non-covalent complexes with polymers; **(D)** nanosystem delivered across the cell membrane via endocytosis to release the therapeutic cargo in proximity of a cytosolic target; **(E)** protein-polymer conjugate and protein-peptide conjugate entering the cell via direct translocation/transduction and delivering the therapeutic material to nucleus receptors. Created with BioRender.com.

During the past decade numerous prominent techniques have been proposed for intracellular delivery of proteins (Fu et al., 2014; Bruce and McNaughton, 2017; Ray et al., 2017; Tian et al., 2022), involving physical methods to cross cell membrane, protein chemical modification and protein transport through carriers (Scaletti et al., 2018; Lee et al., 2019; Goswami et al., 2020) or a combination of the three types. Some examples of the strategies proposed in the next sections are depicted in Figure 1.

In most cases, model proteins have been tested rather than more expensive therapeutic proteins, which are often difficult to track both *in vitro* and *in vivo*. Fluorescent albumin and IgG antibody (Tian et al., 2013; Sarker et al., 2014; Liew et al., 2020; Barrios et al., 2022; Davis et al., 2022), (enhanced) green fluorescent protein (GFP) (Fuchs and Raines, 2007; Kaczmarczyk et al., 2011; Sarker et al., 2014; Zuris et al., 2015; Kube et al., 2017; Liew et al., 2020; Davis et al., 2022), streptavidin (Shi et al., 2017; Davis et al., 2022), horseradish peroxidase (DePorter and McNaughton, 2014), lysozyme (Tian et al., 2013), and ovalbumin (Goswami et al., 2023) are among the typical model proteins used.

### 3.1 Physical membrane crossing methods

Most of the physical approaches for overcoming cell membrane deal with chemical (Stewart et al., 2018) membrane disruption (Mukherjee et al., 2018) or perforation (Chen N. et al., 2022). Although membrane perforation with electroporation (Mukherjee et al., 2018) and microinjection (Keppeke et al., 2015; Chen N. et al., 2022) or sonoporation (Togtema et al., 2012) is the most straightforward method for cytosolic delivery, these strategies are highly efficient in *in vitro* studies (Tan et al., 2022), but generally toxic, only suitable for introducing a small number of specific proteins into incubated cells and can hardly be used *in vivo*.

### 3.2 Chemical modifications of proteins

Protein modification strategy directly features protein with membrane-permeable ligands, such as cell penetrating peptides (Dixon et al., 2016; Su et al., 2018), chimeric peptides (Yu et al., 2021), cationic peptides or polymers (Su et al., 2018), amphiphilic polymers (Liu X. et al., 2019) and protein transduction domains (Caffrey et al., 2016). Alternatively, the chemical alteration consists in supercharging the protein with cationic groups (Horn and Obermeyer, 2021). The biomodification success depends on the availability of reactive protein handles, located on their surface as free amino acid sides, including amine, hydroxy and thiol groups, or functional moieties present at the protein termini (Stephanopoulos and Francis, 2011). There are many covalent methods available for the modification of protein reactive groups including click chemistry, oxime/hydrazone chemistry (Lutz and Börner, 2008), and strategies such as grafting-to, grafting-from and grafting-through for bioconjugation of proteins with polymers (Stevens et al., 2021).

The amended proteins are capable of entering the cells via cellular membrane transduction and translocation (Horn and Obermeyer, 2021) or through endocytosis, achieving high cytosolic delivery (Posey and Tew, 2018) by increased membrane affinity. Sometime covalent modification of proteins is also applied with anionic species, such as carboxylic acid (Wang and Yu, 2020), boronic acid (Liu X. et al., 2019), anionic peptides and polymers (Zelikin et al., 2016), or nucleic acids (Eltoukhy et al., 2014) to strengthen their negative charge intensity, and thus increase their binding affinity with suitable positively charged carriers that enhance endocytosis (Lv et al., 2020). However, covalent modifications often result in a distribution of products with different degrees of modification, owing to chemically identical active sites distributed on the protein surface (Horn and Obermeyer, 2021). Protein alteration can be designed to be reversible, via moieties which can be cleaved by intracellular stimuli such as reduction (Qian et al., 2018), reactive oxygen species (ROS) (Liu X. et al., 2019), enzyme (Chang et al., 2019) and endo/lysosomal acidity (Maier and Wagner, 2012), however covalent modifications may alter protein structures and related biofunctions (Zhou et al., 2019; Tan et al., 2022). Moreover, the technique requires complex synthesis and purification procedures which may impede the potential clinical translation (Frokjaer and Otzen, 2005; Stevens et al., 2021). A meaningful example of protein alteration for cytosolic delivery involves charge-conversional modification of cationic lysine surface moieties by cyclic anhydrides (Obermeyer et al., 2016; Zhang M. et al., 2020; Tao et al., 2020), which is pH-reversible at late endosomal pH. For instance, IgG was modified with citraconic anhydride to encapsulate it in pH-sensitive polyion micelles, capable of transferring active IgG to the nuclear envelope (Kim et al., 2016). Esterification of carboxylic acid groups of aspartate and glutamate simultaneously decrease negative charge and increase hydrophobicity, promoting direct protein translocation across the cell membrane (Sangsuwan et al., 2019).

Stable and simultaneously reversible conjugation is critical to translocate proteins across a cellular membrane and release them without losing activity (Dutta et al., 2021). Liu B. et al. (2019) developed a click chemistry approach for generating functional polymer–protein conjugate as nanoassemblies of different sizes and isoelectric points, which release in response to three different stimuli: presence of ROS, reducing environment, and pH variations. Arylboronic acid was employed for lysines modification, given the possibility of inserting a stimuli-responsive linker in the polymer-protein conjugate, required for a residue-free release (Stephanopoulos and Francis, 2011). They successfully delivered ribonuclease A (RNaseA) via endosomal escape, employing hydrogen peroxide (H_2_O_2_) as the stimulus to reverse the bioconjugation. Similarly, Dutta et al. (2021) designed a self-immolative polymer presenting activated carbonate moieties for covalent self-assembly with the lysines displayed on antibodies surface. The reactive side-chain functionalities were responsive to redox stimuli, and the encapsulated antibodies were released preserving their biological activity. However, slow macromolecular reaction kinetics due to the high acid dissociation constant (pKa) of lysine amines (Koniev and Wagner, 2015), incomplete reactivity of activated carbonate groups with lysines (Dutta et al., 2017), and competitive hydrolytic degradation of polymer, are some of the major obstacles for large biomacromolecules conjugation such as antibodies (Dutta et al., 2021). Considerable attention has been given to enhancing the endosomal escape ability of nanocarriers by incorporating pH-buffering (Lee et al., 2021), membrane-disturbing (Han et al., 2021) or fusogenic (Nishimura et al., 2014) materials. pH-responsive polymeric micelles were designed to promote electrostatic and covalent interactions with anti-nuclear pore complex antibodies (Chen P. et al., 2022). This design reached selective delivery into the cytosol and subsequent nucleus targeting was achieved in cancer cells, rather than non-cancerous cells, both *in vitro* and *in vivo*.

### 3.3 Non-covalent assembly of proteins and carriers

Alternatively, proteins could be transported by carriers through physical encapsulation or complexation. The protein cargoes can be loaded into the inner aqueous/hydrophilic cavities or pores (Tang et al., 2017; Wang and Yu, 2020) of synthetic nanocarriers (Qin et al., 2019), such as liposomes (Wang et al., 2016), polymers (Zhou et al., 2019), polymersomes (Jiang et al., 2018), organic or inorganic nanoparticles (Leader et al., 2008; Zelikin et al., 2016; Lee et al., 2019; Zhang S. et al., 2020), and nanogels (Dutta et al., 2017). These nanomaterials allow intracellular delivery of native proteins without any chemical modification, preventing denaturation (Dutta et al., 2017). This approach is generally suitable for *in vivo* applications (Lv et al., 2020), although it often requires complex syntheses and purification processes with low protein loading efficiency (Liu et al., 2022). On the other hand, protein-based nanocomplexes can be formed via non-covalent interactions with polymers, functionalized nanoparticles, peptides, and lipids. Amino acid residues may interact via salt bridge, boronate-nitrogen (Liu X. et al., 2019; Liu et al., 2022) or metal-nitrogen (Ren et al., 2022) coordination interactions, electrostatic forces (Rui et al., 2019), inter-macromolecular ionic, hydrophobic (He et al., 2019), and hydrogen-bond interactions (Lv et al., 2020). Such assemblies should provide stability during cell membrane penetration and protein release (Yu et al., 2018), via reversible binding (Stevens et al., 2021). They are obtained via simple mixing under mild aqueous conditions, avoiding complex purification steps, without altering the proteins native functions (Posey and Tew, 2018; Lv et al., 2019; Lv et al., 2020; Pasut, 2014). While chemical modification is often limited by the vast heterogeneity in composition, structure, and stability of proteins, non-covalent strategies can be applied to a wide variety of protein cargoes (Posey and Tew, 2018).

In the last years, different nanocomplexes formed via simple self-assembly have been developed (Liu X. et al., 2019; Lv et al., 2020; Wang and Yu, 2020). Hyperbranched polymer with phenylboronic acid (PBA) was developed to coordinate with protein cargoes (Liu et al., 2022), and degrade by over-produced H_2_O_2_ in cancer cells, releasing the proteins (BSA and a monoclonal antibody). Following a similar idea, boronated polymers formed a complex with proteins via nitrogen-boronate coordination and ionic interaction (Yan et al., 2022). Promising cytosolic delivery of cargo proteins and peptides was achieved with maintained bioactivity (Liu X. et al., 2019; Lv et al., 2020). Relying on the same principles, guanidyl groups can strongly bind the residual moieties of protein by a combination of salt bridge and hydrogen bonding interactions. When grafting guanidyl ligands onto nanoparticles or polymers at a high ligand density, the multivalent effect of guanidyl groups allows efficient protein binding (Hatano et al., 2016) and endocytosis (Stanzl et al., 2013; McKinlay et al., 2016). Lv et al. (2020) synthesized guanidyl-grafted polyethylenimine (PEI) to form positively charged nanoparticles with BSA, for an efficient cell membrane penetration. Protein delivery systems poorly performing in serum-containing media were improved by introducing carbamoylated guanidine-containing polymers (Barrios et al., 2022), by chemical modification with fluorous ligands (Zhang et al., 2018) and zwitterionic moieties (Wu et al., 2019), thus decreasing the positive charge density of the nanocomplex (Rui et al., 2019). A rational guanidine modification approach also enhanced the efficiency of proteins delivery in serum-containing media (Li et al., 2015; Keller et al., 2016; Davis et al., 2022). Tan et al. (2022) proposed boronate-decorated poly-L-lysine (PLL) to efficiently deliver cargo proteins into living cells. Positively charged PLL spontaneously form complexes with negatively charged proteins (Abbas et al., 2017). These nanoparticles can release proteins by intracellular ROS after internalization, with maintained activity and minimal toxicity. Amphipathic poly-b-peptides (Pbps), with designable structures, controllable molecular weights, and proteolysis resistant properties, were also investigated for protein delivery (Ren et al., 2022). Pbps amphipathic and positively charged structures promote non-covalent interactions with proteins and membrane disruption (Tezgel et al., 2017), showing successful delivery of EGFP into osteosarcoma cells.

## 4 Discussion

Significant progress has been made in the field of intracellular delivery in recent years, however the clinical applications of protein drugs are still limited by their real delivery efficacy, stability, and intracellular activity. Therefore, research is moving in various directions with the aim of identifying more appropriate delivery tools. The delivery of proteins into cells is challenging due to two major requirements: efficient uptake and rapid cytosolic delivery without being trapped in the endosomes. Many research efforts have been made regarding protein conjugation with cell-penetrating peptides, and more recently with multifunctional chimeric peptides, which can be designed to accomplish different tasks during cellular uptake and endosomal escape. Other methods for the delivery of purified proteins include protein chemical modification and resurfacing approaches. These methods often need to overcome the limits of toxicity and possible immune activation. Nanocarrier-based protein delivery approaches, such as liposomes, polymer-based nanocarriers, and nanoparticles, are attractive due to the tunable properties of the nanomaterials. It is important to consider additional obstacles such as the rapid enzymatic degradation of therapeutic proteins in the bloodstream and potential immune system responses (Moncalvo et al., 2020). Meanwhile, a significant effort has been dedicated to the design of engineered proteins that can be used to modulate intracellular targets (Miersch and Sidhu, 2016). Co-delivery of protein and nucleic acids has also been examined in the context of targeted genomic editing (Bruce and McNaughton, 2017). Moreover, new intracellular targets within subcellular compartments may be identified for a therapeutic use (Fasciani et al., 2022). Delivery of transcription factors also holds the potential to revolutionize the biomedical field (Ulasov et al., 2018), although the major challenge lies in the delivery process, as it requires proteins transport not only across the cell membrane and the endosome, but also into the nucleus, which represents an additional barrier to overcome.

The field of intracellular protein delivery is still a relatively young area of research and further advancements in this field will require the integration of chemistry, materials science, formulation science, nanomedicine, and biomedical engineering. Enabling therapeutic proteins to access and target intracellular receptors has enormous potential for improving human health and fighting diseases, as well as for gaining knowledge in this significant area of research.
